# Foreign Body Infection Models to Study Host-Pathogen Response and Antimicrobial Tolerance of Bacterial Biofilm

**DOI:** 10.3390/antibiotics3030378

**Published:** 2014-08-21

**Authors:** Justyna Nowakowska, Regine Landmann, Nina Khanna

**Affiliations:** 1 Infection Biology, Department of Biomedicine, University and University Hospital Basel, Hebelstrasse 20, 4031 Basel, Switzerland; E-Mail: justyna.nowakowska@unibas.ch; 2 Vice Dean Medical Faculty University Basel, Klingelbergstrasse 61, 4056 Basel, Switzerland; E-Mail: regine.landmann@unibas.ch

**Keywords:** foreign body infection, mouse, staphylococcus, catheter abscess model, tissue cage model

## Abstract

The number of implanted medical devices is steadily increasing and has become an effective intervention improving life quality, but still carries the risk of infection. These infections are mainly caused by biofilm-forming staphylococci that are difficult to treat due to the decreased susceptibility to both antibiotics and host defense mechanisms. To understand the particular pathogenesis and treatment tolerance of implant-associated infection (IAI) animal models that closely resemble human disease are needed. Applications of the tissue cage and catheter abscess foreign body infection models in the mouse will be discussed herein. Both models allow the investigation of biofilm and virulence of various bacterial species and a comprehensive insight into the host response at the same time. They have also been proven to serve as very suitable tools to study the anti-adhesive and anti-infective efficacy of different biomaterial coatings. The tissue cage model can additionally be used to determine pharmacokinetics, efficacy and cytotoxicity of antimicrobial compounds as the tissue cage fluid can be aspirated repeatedly without the need to sacrifice the animal. Moreover, with the advance in innovative imaging systems in rodents, these models may offer new diagnostic measures of infection. In summary, animal foreign body infection models are important tools in the development of new antimicrobials against IAI and can help to elucidate the complex interactions between bacteria, the host immune system, and prosthetic materials.

## 1. Introduction

Among the early and late complications of medical implants, implant-associated infection (IAI) is one of the most serious that is associated with a high morbidity [1]. The average rate of IAI ranges between 2% to 40% depending on the type of surgical implant [2]. These infections occur either perioperatively, by direct bacterial contamination during surgery or wound healing, or by the haematogenous route through blood or lymph [1]. More than 50% of IAI is caused by staphylococci (*Staphylococcus* (*S.*)* aureus* and *S. epidermidis*), followed by streptococci (7%) and *Propionibacterium* spp*.* (6%). Gram-negative bacilli, enterococci, and polymicrobial infections are less frequent (less than 5% each) [3,4]. Over the last four decades, methicillin resistant *S. aureus* (MRSA) has created additional therapeutic challenges. A frequency of 1% to 12% has been reported for MRSA nasal colonization [5], which is associated with a four-fold increased risk of infection [6]. Importantly, it has been estimated that in a high endemicity setting more than half of surgical-site infections due to staphylococci can be caused by MRSA [7].

An important feature of IAI is that the presence of a foreign body increases the susceptibility to staphylococcal infection by at least 10,000-fold [8,9]. Hence, these infections can arise from only a few bacteria inoculated locally during surgery or during bacteraemia. They remain local, but if infection becomes chronic, the implant has to be removed or replaced for healing.

The main reason for persistence of IAI is the microbial ability to form biofilm, which allows bacteria to stay in a metabolically quiescent state. Thus, biofilm governs bacterial recalcitrance to both antimicrobials and host response. As the decreased antimicrobial susceptibility is not drivenby acquisition of any resistance genes and biofilm-embedded bacteria are isogenic with their planktonic antibiotic-susceptible counterparts this phenomenon is referred to as bacterial tolerance. Indeed, proteomic and RNA profiling studies have shown altered gene expression patterns in staphylococcal biofilms, indicating metabolic adaptation [10]. Although the molecular details of biofilm development have been thoroughly investigated, the exact mechanism of its antimicrobial tolerance remains still elusive. 

There are conventionally four stages in biofilm formation distinguished. Initial adherence of planktonic bacteria is facilitated by non-specific and specific interactions, the latter being driven by adhesion to implant-deposited host matrix proteins. Staphylococci developed a variety of adhesins binding those proteins collectively designated as “microbial surface components recognising adhesive matrix molecules” (MSCRAMMs) [11]. Next stages consist of intercellular aggregation and accumulation, maturation, and dispersal of biofilm [11,12,13]. The biofilm matrix can be composed of polysaccharide intercellular adhesin (PIA), the production of which is mediated by the *ica-*locus encoded enzymes, of fibronectin-binding proteins, other large proteins and extracellular DNA [12,13,14]. The expression of those components is governed by four major transcriptional regulators, which are the quorum sensing systems *agr* and *luxS*, as well as *sarA* and the stress sigma factor σ^B^. They interact in a complex network and have variable effects in *S. aureus* and* S. epidermidis*. Environmental factors like oxygen and glucose levels contribute as well to the formation, maturation and dispersal of biofilms [12,15,16]. The growing bulk of biofilm acquires its characteristic three-dimensional architecture during the maturation phase when the fluid-filled channels are formed and this step is substantially governed by phenol-soluble modulins (PSMs) [17,18]. In the final phase bacteria are detached from biofilm, which is facilitated by the accessory gene regulator (*agr*) quorum sensing system and involves PSMs. New niches are colonized and thereby spread of infection occurs [17]. PSM have recently been postulated as potential target for the treatment of *S. aureus* infections [19,20].

Importantly, the *in vitro* biofilm models can differ greatly from the *in vivo* situation where biofilms developed multiple strategies to skew host immune response [21,22,23,24,25,26]. Accordingly, macrophage exposure changed biofilm gene expression profile [25]. We, and others, have shown differential transcription patterns of biofilm regulators in animal models, human infection and *in vitro* [27,28,29]. Altogether, only the *in vivo* systems enable understanding the entire complexity of biofilm-mediated infection.

As a consequence of the biofilm-induced bacterial tolerance antimicrobial treatment of IAI remains challenging. To overcome this tolerance, antimicrobials need to penetrate the biofilm and act on adherent stationary phase-like bacteria. Of note, some antibiotics, e.g., vancomycin and daptomycin, are able to penetrate the biofilm but eventually fail to eradicate the adherent bacteria [17,30]. Thus far, most of the known antibiotics are dependent on the metabolic status of bacteria hindering the eradication of biofilm-embedded quiescent pathogens. The only antibiotic with a proven activity against metabolically inactive staphylococci in IAI is rifampicin [31]. However, due to a rapid emergence of resistance, rifampicin has to be combined with other antibiotics [32,33]. The emergence of resistant bacteria (*i.e.*, MRSA, vancomycin resistant *S. aureus* and methicillin resistant *S. epidermidis*) creates additional challenges, as resistance is associated with a poorer response to therapy. Therefore, novel anti-biofilm agents, such as the acyldepsipeptide (ADEP4) [34], as well as antifouling and antimicrobial implant coatings [35] are under investigation. Altogether, despite proven efficacy of some antibiotics against adherent and metabolically inactive bacteria, antimicrobial therapy of biofilm-mediated infections alone is unsuccessful probably due to the magnitude of the formed biofilm. The biofilm must, therefore, be either removed by surgical debridement or by implant replacement and additionally treated with antibiotics [33].

A further important reason for the persistence of staphylococcal biofilm on foreign bodies is its recalcitrance to host immune responses [36]. Contact with implant surface induces impaired granulocyte functions, including reduced bactericidal activity, impaired oxidative metabolism and spontaneous granular enzyme release [37,38]. Interestingly, human PMNs recovered from patients with osteomyelitis exhibited highly activated phenotype with preserved production of superoxide but impaired chemotactic abilities [39,40]. Moreover, biofilm burden seems to be also dependent on macrophage proinflammatory responses highlighting mutual influence between host cells and biofilm [21]. Accordingly, it has recently been shown that the myeloid-derived suppressor cells (MDSCs) decreased the proinflammatory attributes of monocytes/macrophages and thereby contributed to the chronicity of *S. aureus* biofilm [41]. Finally, the adaptive immune response provided by T helper 2 (T_H_2) and regulatory T-cells (T_reg_), but not T_H_1 and T_H_17, were associated with protection against MRSA biofilm [24].

Taken together, IAI belongs to the leading infections in today’s medicine. To better understand the biofilm antimicrobial tolerance, host response and molecular pathogenesis as well as to develop effective antimicrobials for these infections adequate animal models are needed. Depending on the question to study, different foreign body infection models can be used.

## 2. Subcutaneous Catheter Model

### 2.1. General Aspects of the Subcutaneous Catheter Model

The subcutaneous catheter model is a static model in which an abscess and inflammatory cell recruitment can develop. This model is quite straightforward and less labor-intensive than the later described tissue cage model and is very suitable to study short- and long-term *in vivo* biofilm formation.

### 2.2. Catheter Infection Model in the Mouse

This model has been used over the last two decades by different groups to study the host immune response against biofilm, bacterial virulence factors and treatment of IAI. For example, in a subcutaneous catheter in the mouse [42] *S. epidermidis* infections occurred more often with a biofilm-*ica-*positive than with a biofilm-negative strain and *S. epidermidis* wild type (*wt*) grew more strongly in competitive infection than the *ica^−^* mutant [36]. In a study on infection with a bioluminescent *S. aureus* the efficacy of a four-day rifampicin treatment upon an established biofilm was well documented with this non-invasive method [43].

#### 2.2.1. Technique

**Figure 1:**
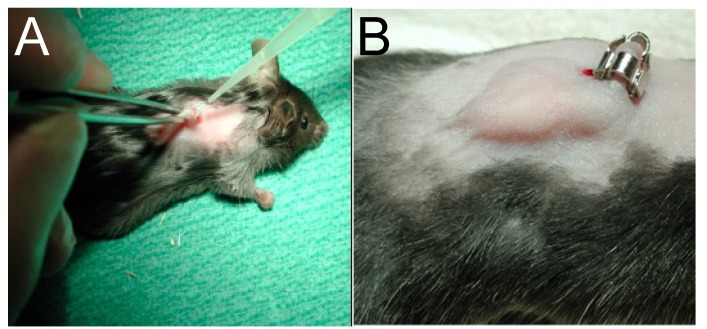
Catheter infection model. (**a**) Infection of catheter; (**b**) Abscess formation after 7 days with *S. aureus* 113 (inoculum 1 × 10^4^ CFU/catheter).

A 3–4-mm incision is made 1–1.5 cm lateral to the spine of 10- to 14-week old C57BL/6 mice, and one catheter segment, either sterile or pre-incubated with a defined inoculum of bacteria (10^4^ to 10^8^ colony-forming units (CFU) for *S. aureus* and *S. epidermidis*), is inserted subcutaneously (Figure 1). Depending on the bacterial species and the inoculum, an abscess can develop, which can be quantified by the oedema cross-section dimension. After sacrifice, bacteria adherent to the catheter or present in the tissue surrounding the catheter are quantified. This has been done one to eight weeks after infection, depending on the abscess development. Additionally, in the capsule that is formed around the catheter and in the surrounding tissue, the cytotoxicity of an investigated compound, as well as host responses to infection can be assessed.

#### 2.2.2. Assessment of the Host Response in the Catheter Infection Model

We used the catheter model to study the mechanism, by which biofilm protects *S. epidermidis* from clearance by host defense. The complement component 3 (C3) activation and C3b/IgG deposition on *S. epidermidis*, as well as granulocyte-dependent killing of *wt* and *ica*^−^ bacteria were compared. We found an enhanced C3 activation by biofilm-positive *S. epidermidis*, yet a decreased complement deposition. These findings correlated with a stronger survival of* wt* *S. epidermidis* on catheters [36]. This is the first observation regarding the molecular pathophysiology of host defense against biofilm. Importantly, the host response to catheter infection can differ from the later described tissue cage, as the recruitment of neutrophils to the catheter can be limited due to the low number of planktonic bacteria [22]. Indeed, macrophages, but not neutrophils, have recently been shown to play an important role in the controlling of staphylococcal biofilm in the catheter infection model [21].

#### 2.2.3. Assessment of Biofilm Formed by *Pseudomonas aeruginosa*

The catheter model is also suitable to study biofilm of other bacteria such as *Pseudomonas aeruginosa*. The role of cyclic di-GMP regulation in small colony variant (SCV) formation, biofilm production and persistence was investigated with mutants overexpressing a diguanylate cyclase YfiN, responsible for the SCV phenotype. Both in single and competitive catheter infections *wt* bacteria were found to be less persistent after eight weeks despite an initial growth advantage [44].

Altogether, there are numerous applications of the catheter model. Toxicity of antibacterial substances can be assessed in the surrounding tissue, which contains, similarly to the tissue cage model, immune, and stromal cells. 

## 3. Tissue Cage Infection Model

### 3.1. Tissue Cage Model in Different Animal Species

The tissue cage model was first described by Zimmerli *et al*. in 1982 [9]. He established the short-term antibiotic therapy of staphylococcal foreign body infection in guinea pigs [45,46] and was the first to observe the granulocyte defects at the site of a foreign body [37]. Since then guinea pigs have been proven as a suitable model for therapeutic studies of IAI [31,47,48,49]. Guinea pigs present the advantage of a high susceptibility to staphylococcal infection, the infection remains strictly local and pharmaco-dynamics and -kinetics of humans can be simulated [50] (Table 1). A drawback is the intolerance of guinea pigs to prolonged antibiotic treatment, as well as to betalactams [50]. Although rats tolerate these antibiotic treatments, they have a 100-fold lower susceptibility to staphylococci. Thus, unless very high inocula are applied, a high proportion of these animals spontaneously clear staphylococcal implant infection [51]. The availability of genetically engineered mice, their susceptibility to staphylococcal infection, their tolerance to antibiotics and advanced animal imaging facilities, allowed mice to become an attractive alternative to study pathogenesis and therapy of IAI using tissue cages. 

**Table 1:** Comparison of orthopaedic and subcutaneous foreign body infection models.

	Orthopaedic Models	Tissue Cage Model			Catheter Abscess Model	Ref.
**Animal Species**	rabbit/sheep/rat/mouse/guinea pig/chicken/dog/pig/goat	guinea pig	Rat	mouse	mouse	[35,52]
**Labor intensity**	+++	+++ §	++	++	+	[50,53]
**Large scale experiments**	no	nd	nd	yes	yes	[54,55]
**Localization**	bone	sc	sc	sc	sc	[56]
**Susceptibility to staphylococcal infection**	species-dependent	yes	no	yes	yes	[35,50,52]
**Antibiotic tolerance (long-term treatment)**	species-dependent	no	yes	yes	yes	[50]
**Use of transgenic animals**	nd	nd	nd	yes	yes	[57]
**Imaging**	yes	nd	yes	yes	yes	[35,56,58,59,60,61,62,63,64,65]
**Bacterial virulence factors**	only after sacrifice	yes	no	yes	yes	[18,27,35,44,57,66,67,68,69,70,71,72,73,74,75,76]
**Host immune response**	yes	yes	yes	yes	yes	[21,36,37,54,58,62,63,77,78,79,80]
**Osseointegration**	yes	no	no	no	no	[35]
**Various implant materials/coatings**	yes	nd	nd	yes	yes	[38,81,82,83,84,85]
**Repeated assessment during experiment:**						
**Cytotoxicity on eukaryotic cells**	no	nd	nd	yes	no	[82,86]
**Pharmacokinetics (PK) at the infection site**	no	yes	yes	yes	no	[9,30,50]
**Pharmacodynamics (PD)**	no	yes	yes	yes	no	[30,32,35,86,87,88,89,90,91]
**Similarity to human disease:**							
**Localized infection**	yes	yes	yes	yes	no	[35,50]

nd: not defined, sc: subcutaneous. ^§^ For every procedure 2 persons are needed.

### 3.2. Tissue Cage Infection Model in the Mouse

#### 3.2.1. Technique

**Figure 2:**
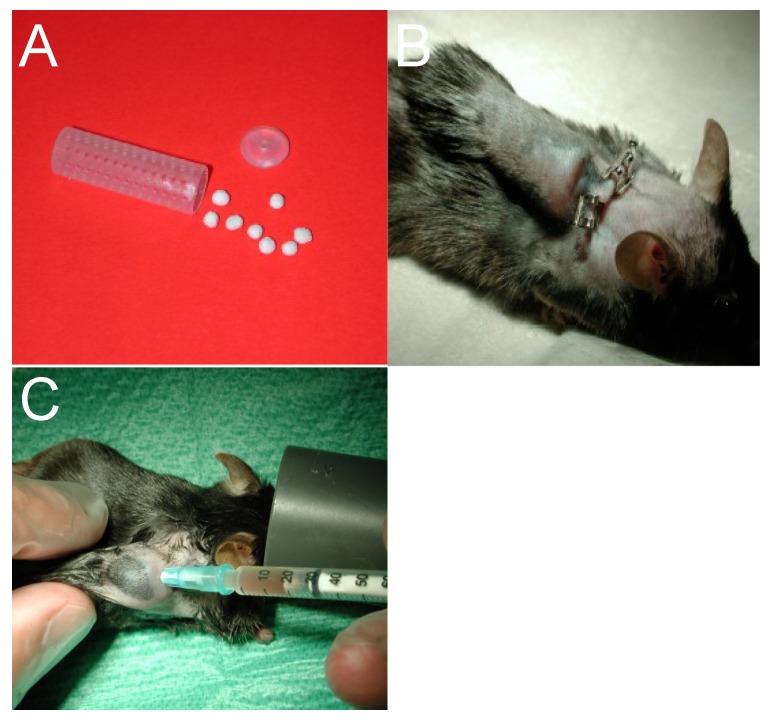
(**a**) Teflon cage with glass sinter beads; (**b**) Mouse ten days after implantation; (**c**) Aspiration of tissue cage fluid under isofluran anaesthesia.

Cylindrical tissue cages (8.5 × 1 × 30 mm, volume 1.9 mL) [50] are manufactured from Teflon or from any type of metal or alloy [83]. The wall of each cage is perforated with 130 regularly spaced 1.0-mm holes. A hole of 2 mm in diameter is placed both in the Teflon lid and in the bottom of the cage. Importantly, the design of the tissue cages can be changed according to specific experimental requirements. To increase the surface area, cages can be filled with beads from sinter glass or from any material. Conversely, pieces of plastic catheter have also been used in place of beads [27]. Thus, the cages can be filled with various materials, but, thus far, data on the presumable impact of those on the biofilm formation are missing. Cages are implanted subcutaneously into the back of anesthetized 12- to 15-week-old C57BL/6 mice (Figure 2a,b). Bacteria are injected directly into the cage either perioperatively or around 14 days postoperatively. In contrast to guinea pigs, *S. epidermidis* should be injected only perioperatively as it can be spontaneously cleared if injected postoperatively in mice [83]. *S. aureus* needs to be injected post-operatively after wound healing to avoid the risk of surgical site infections with deep abscesses. Sterility before infection and the establishment of an infection are confirmed by quantitative culture of tissue cage fluid (TCF). The infection with and without subsequent therapy is usually followed for 14 days, however, mice tolerate, as well, a prolonged infection and antimicrobial treatment without systemic signs [92]. The inflammation remains localized and animals in general do not develop bacteraemia. 

The load of planktonic bacteria and the local host immune response to infection are assessed by repetitive puncture of TCF (Figure 2c). TCF resembles the extracellular fluid with about 50% of the serum protein concentration, similarly to noninflammatory interstitial fluid [9].

#### 3.2.2. Assessment of Virulence of Bacterial Species

The inoculum necessary for induction of a persistent infection in tissue cages of C57BL/6 mice is assessed by identifying the minimal infective dose (MID) of the investigated bacterial species or strains. The MID is an indicator of staphylococcal virulence in this model. For *S. aureus* it ranges between approximately 5 × 10^2^–5 × 10^3^ CFU/cage [9,30,86]. In contrast, the MID of *S. epidermidis* is much higher, namely above 10^6^ CFU/cage, and spontaneous clearing occurs, if bacteria are not injected during the perioperative period [83]. In infections with isogenic mutants of staphylococci, which have specific deletions of virulence genes, a higher MID may be required. This was for example shown for the *S. aureus dlt^−^* mutant, which manifested a 100-fold higher MID than the parental *wt* strain. This mutant expresses non-alanylated lipoteichoic acid, which renders the surface charge of the bacterial cell wall more negative, and, thus, more susceptible to cationic antimicrobial peptides. Therefore, the *dlt*^−^ mutant is more easily cleared unless the infective dose is increased [57]. On the other hand, the virulence of *S. aureus* mutants lacking the *ica* gene responsible for polysaccharide-mediated biofilm formation, has not been attenuated in the tissue cage model [70]. Even in competitive infection studies with simultaneous inoculation of both *wt* and *ica*^−^ *S. aureus*, the* ica* mutant did not grow less efficiently than the *wt* [27]. These observations were surprising, since *ica* expression is considered one of the crucial contributors to staphylococcal biofilm, which is generally considered as the major virulence factor in IAI. Indeed, in contrast to the* S. aureus* counterpart,* ica* mutants of *S. epidermidis*, showed reduced virulence in the tissue cage model [27] and in catheter-associated infections both in rats [76] and in mice in studies from others and our own group [42,83]. Thus, these results illustrate that conclusions on virulence in IAI models can only be applied to the particular bacterial species and the exact model used in the given investigation, *i.e.*, for *S. epidermidis* biofilm plays a more significant role in virulence than for *S. aureus*, which has multiple factors mediating adherence [93,94]. 

The tissue cage can be considered as a closed *in vivo* system, in which any bacterial species is exposed to host phagocytes. In that context the model has been shown to be suitable to assess the role of a sialidase in *Capnocytophaga canimorsus* *in vivo*. This commensal bacterium was shown to survive *in vitro* only in the presence of human cells, where it could feed on host glycoproteins using its surface-exposed sialidase. This behavior could also be demonstrated by infection with *wt* but not with sialidase-deficient bacteria in normal and leukocyte-depleted tissue cages [95].

**Figure 3:**
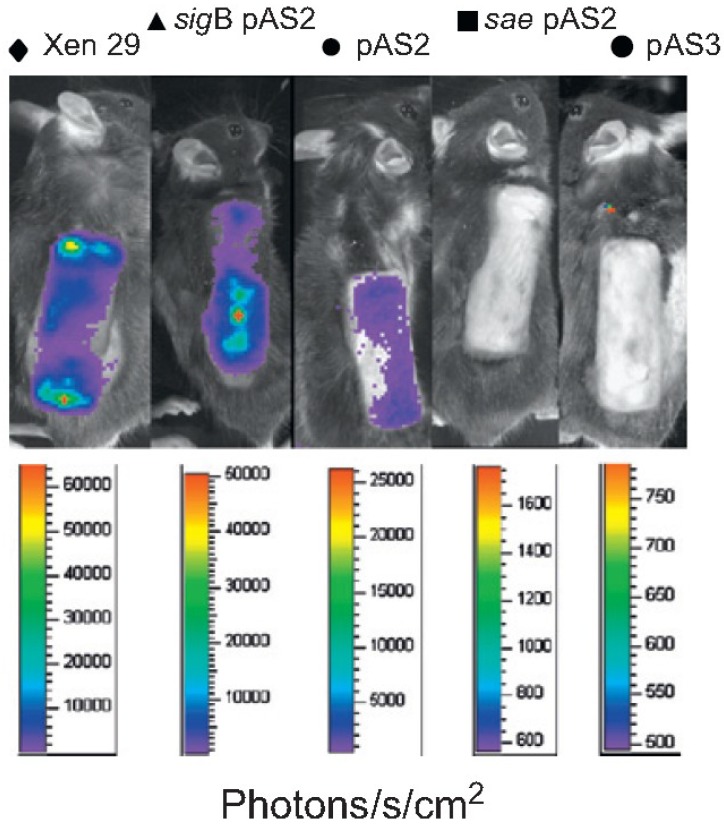
Visualization of *S. aureus hla*^−^ promoter activity using luxABCD integration vector. The *phla*-*lux* construct was introduced *via* a single chromosomal insertion in the *S. aureus* *wt* strain Newman and its isogenic *sae* and σ^B^ regulator mutants. *hla*-Expression was followed in real-time at repeated time points of infection (here, day eight) of a mouse tissue cage using a photon-sensitive camera. The activation of *hla* in the σ^B^-deficient strain and the repression to background levels in a *sae*-deficient strain relative to the *hla*-expression in the *wt* is shown. Bacterial numbers did not differ among the different strains [64].

Another interesting feature to study biofilm *in vivo* is to combine it with bioluminescence imaging. A chromosomally expressed *lux* operon in *S. aureus* renders bacteria visible in a CCD camera and allows close observation of the bacterial load during infection [96]. A more sophisticated application of this technique is to follow promoter activity of a virulence factor in *S. aureus.* For this aim we transduced a specific promoter-regulated *lux* operon into *wt* or isogenic mutant of *S. aureus* [64]. We could demonstrate an increasing activity of the *hla* promoter during eight days of a tissue cage infection and its modulation by transcriptional regulators σ^B^ and *sae* [64] (Figure 3). Nevertheless, the targeted bioluminescence is limited by its relatively low sensitivity due to the single copy of the gene in question.

#### 3.2.3. Assessment of Host Defense in the Tissue Cage

TCF is an extracellular fluid containing myeloid cells as innate defense system. Strikingly, granulocytes in the neighborhood of a tissue cage display weakened functions, including bactericidal activity, oxidative burst, phagocytosis and spontaneous loss of granules [37]. To investigate in depth the role of leukocyte subpopulations in the defense against tissue cage infections, experiments in leukocyte- or granulocyte-depleted mice can be performed. Furthermore, the mechanisms of host defense in this infection model can also be unravelled in knockout mouse strains with specific deficiencies of the innate immune system. As an example, we could show that *dlt^−^* bacteria, which were cleared in *wt* mice, proliferated in TLR2-deficient host, thus identifying a role of TLR2 in murine immune defense against bacteria expressing unalanylated teichoic acids [57].

#### 3.2.4. Assessment of Antibiotic Resistance *in Vivo*

Little is known about the evolution of antibiotic resistant staphylococci during an infection. The tissue cage model provides the opportunity to investigate an antibiotic-resistant staphylococcal strain during the course of infection over a prolonged period of time (two to four weeks). Stability of genetic and phenotypic changes, which characterize the resistance, can be repeatedly evaluated in the treated or untreated tissue cage. This approach was used to investigate glycopeptide (teicoplanin)-intermediate resistance in *S. aureus* (GISA). This resistance arises from prolonged glycopeptide exposure and is the result of multiple unknown mutations leading to a common phenotype of GISA. Gene expression and phenotype were followed in isogenic GISA and *wt* strains without antibiotics in the tissue cage. Interestingly, teicoplanin resistance posed a fitness burden on *S. aureus*, which resulted in a negative selection *in vivo* with restoration of fitness incurring the price of resistance loss [97].

#### 3.2.5. Pharmacokinetic (PK) Studies, Pharmacodynamic (PD) Properties and Efficacy

While many *in vitro* tests can give hints on antimicrobial properties of new drugs, mouse models have been developed in order to assess their efficacy *in vivo*. This is of great interest, since *in vivo* and *in vitro* results of transcriptional regulators and biofilm biosynthesis genes were found to differ [27,28,29]. *In vitro* growth curves, minimal inhibitory and bactericidal concentrations (MIC and MBC, respectively) for logarithmic and stationary growth-phase bacteria are determined beforehand for a drug of interest.

Because the goal of antibiotic treatment studies in animals is to mimic the conditions in humans, PK of the drugs has to be adapted accordingly. PKs reflecting those in humans have been determined for daptomycin with 30 mg/kg [30], 40 mg/kg [30], and 50 mg/kg [98] applied intraperitoneally (i.p.) once per day [30], vancomycin 200 mg/kg two times per day, levofloxacin 150 mg/kg three times per day and ciprofloxacin 100 mg/kg twice per day in our mouse tissue cage model [99]. For practical reasons, a once- or twice-daily regimen is chosen in most experiments. The PKs of mice differ greatly from other animals, such as rats and guinea pigs [50]. 

Antibiotics can be applied i.p., subcutaneously, intramuscularly, orally and directly into the TCF. We mainly apply the antibiotics i.p. or into the TCF, which is of particular interest for new compounds that have poor or unknown *in vivo* PK profile. Thus, small cost-effective *in vivo* PK and toxicity studies can be done in the mouse tissue cage model with minimal compound requirement and multiple endpoints.

Treatment studies in mice have been adapted according to the previously described guinea pig model [50]. Twenty-four hours upon injection of a staphylococcal MID, the establishment of infection is confirmed after TCF sampling. These short-term infections and low inocula are used, as it is well known that antibiotic treatment does not eradicate chronic biofilm infection. Most of the treatment studies are thus four days. Treatment duration could be prolonged but has been adapted according to the guinea pigs that do not tolerate longer antibiotic exposure. On day one and four, TCF is collected to quantify planktonic bacteria and the animals are sacrificed. As the ultimate goal of antimicrobial treatment is to eradicate not only planktonic but all adherent bacteria on an implant, the presence of adherent bacteria is also determined. Tissue cages are removed under aseptic conditions and incubated in broth for 48 h, followed by assessment of bacterial growth. A positive culture is defined as a treatment failure. The efficacy of treatment against adherent bacteria is expressed as the cure rate (in percent), defined as the number of cages without growth divided by the total number of cages in the individual treatment group. Depending on the type of antibiotic used, to avoid the carry-over effect and false negative culture animals can be kept more than four day after drug withdrawal. Using this procedure, it became evident that daptomycin is not efficacious against adherent bacteria. The tolerance of adherent *S. aureus* to daptomycin was not related to biofilm, but was likely due to enhanced membrane stability during adherence and could be reverted by addition of Ca^2+^ ions [30].

In a recent study, we investigated the antimicrobial activity and mode of action of a serrulatane compound, 8-hydroxyserrulat-14-en-19-oic acid (EN4), a diterpene isolated from the Australian plant *Eremophila neglecta*. *In vitro* EN4 elicited antimicrobial activity toward various Gram-positive bacteria in logarithmic, stationary growth phase and embedded in biofilm. Additionally, EN4 was also cytotoxic against eukaryotic cells. *In vivo* however neither bactericidal nor cytotoxic effects were present, indicating an inhibition of its activity. Inhibition assays revealed that this was caused by interaction of EN4 with albumin [86].

#### 3.2.6. Cytotoxicity of New Antimicrobial Compounds against Host Cells 

In the evaluation of new anti-infective compounds, the therapeutic window is an early predictor of success or failure in drug development. Leukocyte viability in the tissue cage is an indicator of *in vivo* drug cytotoxicity. Mechanistic aspects of eukaryotic cell death can be distinguished *ex vivo* by flow cytometric analysis of apoptosis and necrosis. The evaluation of efficacy and at the same time toxicity from one sample is particularly important for compounds with the same mechanism of action on eukaryotic and prokaryotic cells, such as silver. Indeed, silver ions, which undergo a revival as antibacterial compounds, block respiratory enzymes both in human cells and in bacteria. Thus, the therapeutic window is likely very small and the silver concentration the eukaryotic cells are exposed to must be limited, e.g., by applying slow-release compounds. We have tested *in vivo* the bactericidal activity of silver coordination polymers coated on titanium cages. Indeed, the bactericidal activity on planktonic *S. epidermidis* was paralleled by a transient decrease in leukocyte viability in the cage [82]. However, histological investigation of the surrounding tissue of silver-coated cages including the capsule and the muscle did not show increased inflammation or necrosis compared to uncoated cages [100]. 

#### 3.2.7. Properties of Different Tissue Cage Materials

Tissue cages can be manufactured from Teflon, ceramics or any metal or alloy. This enables investigating antifouling properties and biocompatibility of novel implant materials in preclinical studies. For instance, we found that the metal titanium or steel played a minor role in propensity to biofilm generation or in persistence of staphylococcal infection [83].

## 4. Orthopaedic Implant Infection Models

The main limitation of the subcutaneous foreign body models, such as the described here tissue cage and catheter model, which well simulate extravascular IAI in human settings [38], is that the aspects of osseointegration cannot be addressed. There has been recently published a comprehensive review about the orthopaedic animal models for investigation of IAI [35], which are beyond the scope of this review. Many of these models involve insertion of implants into bones of the lower limb. They are suitable for studies of materials and their interactions with the bone tissue. The most recent models use bacteria-loaded pins inserted into the mouse tibia [23] or bacteria-loaded holes drilled with screws in the rabbit femur [101]. Other models in rabbits and sheep introduce bacteria in cement into the medullary cavity of tibia [61] or during a tibia osteotomy with a locking compression plate [102], respectively. The differences between the orthopaedic and the subcutaneous foreign body models described here are summarized in Table 1. Orthopaedic models allow imaging, bacteriological and histological analyses only after sacrifice. They are not suitable for multiple sampling to test antimicrobial activity, pharmacokinetics, and to investigate the immune response. Furthermore, these models often require sophisticated surgical techniques and high numbers of large animals in order to determine the time course of infection.

A recent study has overcome these drawbacks by using a genetically engineered mouse strain with fluorescent myeloid cells and infection with bioluminescent staphylococci. The authors inoculated the knee joints after placement of a wire implant into the mouse femur. They determined the quantity and localization of both bacteria and neutrophils noninvasively and longitudinally by 3D fluorescence and bioluminescence imaging and they assessed the anatomical bone changes using micro-computed tomography registration. However, the advanced equipment required for analysis does not yet allow application of this method in practice to various animal species and bacterial clinical isolates [63].

In summary, IAI models in bone are of a great interest, as they closely mirror the clinical situation of bone-inserted implants. Nevertheless, they are technically difficult and do not allow an easy assessment of the antibacterial effect and host immune response.

## 5. Disadvantages of Subcutaneous Animal Foreign Body Models

Despite the advantages of these models, they also have some limitations. With the tissue cage and catheter model, only general aspects of host response and biocompatibility can be analyzed. However, specific problems related to bone implants, vascular grafts, or neurosurgical devices cannot be studied. In addition, for PK and PD studies, the special situation of metabolic processes in small animals as compared to humans has to be considered.

## 6. Conclusions

The two described subcutaneous IAI models are well-established long-standing *in vivo* models in which microbiological, pharmacological, immunological, and chemical properties of biomedical implants can be assessed. Both models are easy to perform. Up to 20 animals can be implanted, infected and treated daily by one person. Subcutaneous tissue cages or catheters are well tolerated by mice, even for prolonged periods exceeding one month. The particular advantage of the tissue cage model is the closed system that allows the repeated assessment of the interactions between antimicrobials, host responses and biofilm-forming bacteria *in vivo*. The fact that the cages can be manufactured from any material, which is used in orthopaedic implants, makes the model relevant for pre-clinical application. The cages can be coated with any new compound as local anti-infective or anti-adhesive substance to prevent IAI. The particular advantage of the catheter infection model is its suitability for the molecular *in vivo* studies of biofilm with various Gram-positive and Gram-negative bacteria. Current and future work focuses on the development of implant surfaces with covalently coated or triggered-release antimicrobials to prevent IAI and on new compounds that inhibit the formation of biofilm.
